# Evolution of Pig Fecal Microbiota Composition and Diversity in Response to Enterotoxigenic *Escherichia coli* Infection and Colistin Treatment in Weaned Piglets

**DOI:** 10.3390/microorganisms9071459

**Published:** 2021-07-07

**Authors:** Mohamed Rhouma, Charlotte Braley, William Thériault, Alexandre Thibodeau, Sylvain Quessy, Philippe Fravalo

**Affiliations:** 1 Department of Pathology and Microbiology, Faculty of Veterinary Medicine, Université de Montréal, Saint-Hyacinthe, QC J2S 2M2, Canada; braley.braley@umontreal.ca (C.B.); william.p.theriault@umontreal.ca (W.T.); alexandre.thibodeau@umontreal.ca (A.T.); sylvain.quessy@umontreal.ca (S.Q.); philippe.fravalo@lecnam.net (P.F.); 2 Groupe de Recherche et d’Enseignement en Salubrité Alimentaire (GRESA), Faculty of Veterinary Medicine, Université de Montréal, Saint-Hyacinthe, QC J2S 2M2, Canada; 3 Conservatoire National des Arts et Métiers (CNAM), 292 rue Saint-Martin, 75003 Paris, France

**Keywords:** *Escherichia coli*, ETEC, colistin, 16S rRNA, fecal microbiota, diversity, weaned piglets

## Abstract

The intestinal microbiota plays several important roles in pig health and growth. The aim of the current study was to characterize the changes in the fecal microbiota diversity and composition of weaned piglets following an oral challenge with an ETEC: F4 strain and/or a treatment with colistin sulfate (CS). Twenty-eight piglets were used in this experiment and were divided into four groups: challenged untreated, challenged treated, unchallenged treated, and unchallenged untreated. Rectal swab samples were collected at five sampling times throughout the study. Total genomic DNA was used to assess the fecal microbiota diversity and composition using the V4 region of the 16S rRNA gene. The relative abundance, the composition, and the community structure of piglet fecal microbiota was highly affected by the ETEC: F4 challenge throughout the experiment, while the oral treatment with CS, a narrow spectrum antibiotic, resulted in a significant decrease of *E. coli/Shigella* populations during the treatment period only. This study was the first to identify some gut microbiota subgroups (e.g., *Streptococcus*, *Lachnospiraceae*) that are associated with healthy piglets as compared to ETEC: F4 challenged animals. These key findings might contribute to the development of alternative strategies to reduce the use of antimicrobials in the control of post-weaning diarrhea in pigs.

## 1. Introduction

During the last decade, increasing attention has been paid to the study of the intestinal microbiota of mammals and its relationship to health, well-being, nutrition, and disease [1,2,3]. These studies were facilitated by the development of culture-independent techniques that use the bacterial 16S ribosomal RNA (rRNA) gene as a molecular target to analyse the diversity of a given microbiota [4,5]. It is now accepted that the intestinal microbiota of pigs harbors a large and diverse number of microorganisms that contribute to their health status by stimulating the normal maturation of host tissues, by providing a key non-specific defense, and by contributing to nutritional functions [6]. The total number of bacteria in the pig colon has been estimated to be 1 × 10^10^–1 × 10^11^ per gram of gut content [7]. The community composition and structure of pig gut bacteria is largely determined by factors such as diet, age, genetics, environmental conditions, microbial infection, and antimicrobial exposure [8,9]. Weaning is the most critical phase in a pig’s life due to the sudden social, dietary, and environment changes [10]. Several major changes occur in the composition of piglet microbiota due to the feed and environment modifications at this time [11]. After weaning in healthy piglets, the fecal microbial community is dominated at the phylum level by Bacteroidetes (59.6%), Firmicutes (35.8%), Spirochaetes (2.0%), Proteobacteria (1%), and Tenericutes (1%) [12,13]. During the weaning transition a clear shift in the fecal microbiota from Firmicutes to Bacteroidetes at the phylum level and from *Bacteroides* to *Prevotella* at the genus level has been observed [12], as well as an increasing abundance of *Lactobacillus* [14,15].

During weaning, piglets are predisposed to several microbial infections, particularly those caused by enterotoxigenic *E. coli* (ETEC), the bacterial agent most often involved in post-weaning diarrhea (PWD) [16]. Adhesion of ETEC to the intestinal mucosa of piglets is mediated by F4 or F18 specific receptors; the existence and function of these receptors are crucial for the clinical occurrence of PWD [16]. Because PWD in pig production is characterized by a reduction in feed intake, poor growth rate, diarrhea, and significant mortality [17], it is an economically important disease. There have been several reports that show ETEC infections significantly impair performance, resulting in a reduction in average daily gain and in final body weight of weaned piglets [17,18]. Many studies have also been conducted on the development of gut microbiota in piglets during the pre- and post-weaning periods [11,14,19,20,21,22]; however, studies examining the change in gut microbiota of piglets with clinical ETEC-related PWD remain scarce. It is therefore poorly understood how ETEC infection modifies pig fecal microbiota and if these changes are transient or lasting. 

Furthermore, the administration of antimicrobials, which are often used during the post-weaning period, could impact intestinal microorganism abundance and may cause a severe disruption of the piglet gut microbiota ecosystem [23,24]. Colistin, a polymyxin antibiotic, is widely used for the control of *Enterobacteriaceae* infections in pigs [16,25]. This antibiotic has a narrow antibacterial spectrum with an effect limited to Gram-negative bacteria (GNB) [26]. Colistin sulfate (CS) is the only form of colistin approved in pig production in some countries (other than Canada and the United States) for the control of PWD, being used orally at the dose of 50,000 IU/kg body weight (BW) every 12 h for a period of 3 to 5 consecutive days [17,27]. It is noteworthy that CS has a very low oral bioavailability when administered to pigs [28]. Consequently, a pig’s microbiota is exposed to the majority of the ingested dose of CS, which could potentially disrupt the microbiota composition of the treated animals. In healthy piglet fecal microbiota, the oral administration of CS at the recommended dose was associated with a significant decrease of the *E. coli* population during the treatment period; no other significant perturbation of the fecal microbiota was reported in these samples [29]. However, to the best of our knowledge, the impact of CS on the fecal microbiota of sick piglets has never been studied using high throughput sequencing. Moreover, characterizing the intestinal microbiota of pigs with clinical PWD could provide insight into methods of better controlling this disease and could even help in the development of alternative strategies to the use of antimicrobials in pig production. The main goal of this study was to determine the effect of ETEC: F4 challenge and oral CS treatment on the fecal microbiota composition and diversity of both clinically healthy and challenged piglets. The present study investigates, for the first time, a very common situation occurring in the field during the weaning period, when animals are infected by ETEC strains and simultaneously treated with antimicrobials.

## 2. Materials and Methods

### 2.1. Animals, Experimental Design and Sample Collection

**Figure 1:**
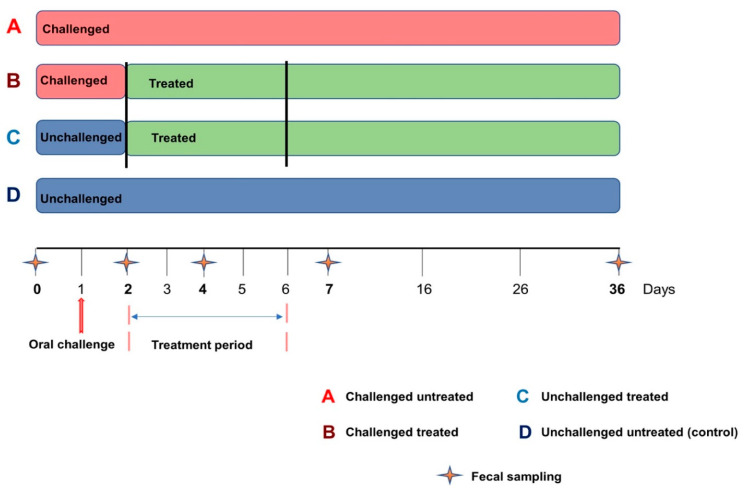
Experimental design. The challenge corresponded to the oral administration of 10^9^ CFU of ETEC: F4 strain to each challenged piglet. The treatment period corresponded to the administration of colistin sulfate at the dose of 50,000 IU/kg twice a day, from Day 2 to Day 6.

It should be noted that the current study was part of a larger project aimed at (1) characterizing the pharmacokinetic parameters of two doses of colistin sulfate (CS) in piglets and studying their therapeutic efficacy on commensal and pathogenic *E. coli*, on *E. coli* resistance development, and on the zootechnical performance (body weight) evolution of piglets in an experimental model of enterotoxigenic *E. coli* (ETEC) infection in weaned pigs [17]; (2) characterizing the fecal presence of ETEC enterotoxin as well as F4 and F18 genes as an indicator of CS efficacy for the treatment of PWD in pigs using the conventional dose of this antibiotic (50,000 IU/kg) [30]; and (3) characterizing the effect of ETEC: F4 challenge and oral CS treatment (conventional dose) on the fecal microbiota composition and diversity of weaned piglets (present study). Only weaned piglets from the second trial of the project (those receiving 50,000 IU/kg of CS) [17] were used in the current study. Briefly, a total of 48 Duroc-Yorkshire-Landrace clinically healthy weaned piglets, 21 days of age, were used to carry out the experiment. Animals were obtained from a local pig producer (Ange-Gardien, QC, Canada) and housed at the biosecurity level 2 agro-environment platform for farm animals at the Faculty of Veterinary Medicine (FVM). Piglets were divided into four groups of 12 pigs each: challenged untreated (group A), challenged treated (group B), unchallenged treated (group C), and unchallenged untreated (group D) (Figure 1). Challenged piglets were housed in the same room (in two different pens), while unchallenged piglets were housed in two separate rooms. After the one week period of acclimatization (at 28 days old), pigs in the challenged groups were orally gavaged with a single dose of 10^9^ CFU of ETEC: F4 strain ECL8559A (O149: LT: STa: STb: F4: Nal^R^) kindly provided by the OIE Reference Laboratory for *Escherichia coli* (EcL, FVM from the Université de Montréal) as previously described [17,30]. Colistin sulfate (Bond & Beaulac Inc., Acton Vale, QC, Canada) was administered orally in 5 mL of water using a polyethylene tube attached to a syringe, at a dose of 50,000 IU/kg twice a day for 5 successive days as previously described [17]. Diarrhea scores, ETEC: F4 fecal shedding, rectal temperature, and body weight were measured and were previously published [17]. It is worth noting that one pig in the challenged treated group died 2 days after the oral challenge and two pigs in the challenged untreated group died at 4 and 6 days after the challenge as previously reported [17]. Individual fecal samples were collected using sterile rectal swabs one day before the challenge (D0) and at 1 (D2), 3 (D4), 6 (D7), and 35 (D36) days after the challenge (Figure 1). It should be stressed here that rectal swab samples from groups C and D (unchallenged piglets) were not collected at D2, because we did not expect a difference in the fecal microbiota composition of these animals between D0 and D2. All rectal swab samples were immediately snap-frozen in liquid nitrogen and stored at −80 °C for microbiota analyses. All animals were euthanized 35 days after the oral challenge.

### 2.2. Genomic DNA Extraction and Purification

It should be mentioned that, after thawing samples, we found that some rectal swabs contained no feces, probably because some animals had diarrhea or because their rectums were empty during fecal sampling. For the present study, we decided to consider only piglets for which we had rectal swabs containing feces for the five sampling times. Considering this requirement, rectal swab samples derived from 28 piglets were selected for genomic DNA extraction in the current study. Groups consisted of 7 pigs each and were classified as challenged untreated (group A), challenged treated (group B), unchallenged treated (group C), and unchallenged untreated (group D). Total genomic DNA was extracted using a standard Phenol-Chloroform method as previously described [31]. DNA concentrations were determined using a Nanodrop^®^ ND-1000 spectrophotometer (Thermo Scientific, Wilmington, DE, USA) and a Qubit^®^ 3.0 broad range assay (Fisher Scientific, Ottawa, ON, Canada) run on a Denovix (Wilmington, DE, USA) fluorometer, respectively. The DNA integrity was determined by agarose gel electrophoresis (concentration of agarose gel: 1.8%, voltage: 100V, and electrophoresis time: 45 min). The DNA samples were then stored at −20 °C for further analysis.

### 2.3. 16S rRNA Gene Sequencing

The 16S rRNA gene V4 hypervariable region-encoding sequences in the genomic DNA for each sample was amplified by PCR using universal primers F515 (5′-GTGCCAGCMGCCGCGGTAA-3′) and R806 (5′-GGACTACHVGGGTWTCTAAT-3′) as described by Zhang et al. [18] and Caporaso et al. [32]. The PCR thermal profile consisted of an initial denaturation of 5 min at 98 °C, followed by 23 cycles of 30 s at 98 °C, 30 s at 55 °C, 3 min at 72 °C, and the final step of 10 min at 72 °C. The quality of the PCR products was assessed by gel electrophoresis (concentration of agarose gel: 1.8%, voltage: 100V, and electrophoresis time: 45 min). Amplicons were barcoded and paired-end sequenced (PE 2 × 250) using an Illumina MiSeq system at Genome Québec. The PCR amplification step included 3 negative controls (sterile water) and a positive control that contained DNA from eight known bacterial DNA with different 16S rRNA gene abundance (18.4% *Lactobacillus*, 17.4% *Bacillus*, 15.5% *Staphylococcus*, 14.1% *Listeria*, 10.4% *Salmonella*, 10.1% *Escherichia*, 9.9% *Enterococcus* and 4.2% *Pseudomonas*) (ZymoBIOMICS Microbial Community DNA Standard (Zymo Research, Irvine, CA, USA)).

### 2.4. Sequence Analysis, OTU Assignment and Diversity Determination

Raw sequencing reads were demultiplexed, quality-filtered and analyzed using Mothur software [33], v.1.43.0 as previously described [31]. Sequences were aligned against SILVA 132 Mothur-formatted reference database. Chimeras were checked and removed using the VSEARCH algorithm. The resulting high-quality reads were clustered into operational taxonomic units (OTUs) with a 97% similarity threshold in Mothur. The OTU taxonomic assignation was performed with the RDP database using a 70% cut-off. Samples were grouped according to the type of oral administration (CS and/or ETEC: F4), by control group, and by the 5 sampling times in the experiment. Further analysis was conducted in RStudio version 1.2.5033 (RStudio: Integrated Development for R. RStudio, PBC, Boston, 2020).

After rarefaction to an even sequencing depth, alpha diversity of the piglet fecal microbiota was analyzed using the observed total number of OTUs (present in each sample) and the Inverse Simpson and the Shannon indices [34]. A Kruskall–Wallis test, with a statistical significance of *p* < 0.05, was used to compare the alpha diversity measures between all groups (A, B, C, and D together) at D0. The same test was also used to perform 2 by 2 comparison between animal groups (A and D, B and C, C and D, and A and B) at the other four sampling times, respectively. 

For the beta-diversity analysis, as a measure of similarity between samples, after a subsampling based on number of sequences per sample as described above, a distance matrix comparing all the samples was created by using Jaccard and Bray–Curtis dissimilarity indices as previously reported [31]. These results were visualized using non-metric multidimensional scaling (NMDS) graphs. The beta diversity was statistically compared using the Analysis of Molecular Variance (AMOVA) test at D0 by comparing the four groups (A, B, C, and D), and at the other four remaining sampling times by doing 2 by 2 comparisons between groups (A and D, B and C, C and D, and A and B).

Species abundances were compared between groups using Multivariate Association with Linear Models 2 (MaAsLin2) [35], using 2 by 2 comparisons at D4, D7, and D36, respectively. For this, species were regrouped at the same taxonomic rank (family and genus). All analyses in MaAsLin2 were performed with the default options. Multivariate association was considered significant between animal groups at *p*-value < 0.05 and a *q*-value < 0.25.

## 3. Results

### 3.1. Sequence Quality 

The V4 region of 16S rRNA genes were sequenced from 119 rectal swab samples (recovered from the four groups during the five fecal sampling times) as well as from three negative (sterile water) and one positive (Mock community DNA) control. It is noteworthy that 7 rectal swab samples (4 from the group A, 1 from group B, 1 from group C, and 1 from group D) were not considered in the fecal microbiota analysis because the bacterial DNA concentration derived from these samples was too low (<1 ng/μL) to allow for sequencing. After removing incorrect and chimeric sequences, more than 8.4 million high-quality reads were generated and clustered into 5445 OTUs and then assigned to 21 phyla, 39 classes, 66 orders, 138 families, and 339 genera. An average of 66,373 sequences per sample were obtained. The negative control contained 12 OTUs (8336 sequences) (Table S1). The positive control (microbial community DNA standard) contained 132,505 sequences and was composed of 16.6% *Lactobacillus*, 15.8% *Staphylococcus*, 14.7% *Bacillus*, 14.3% *Salmonella*, 11.5% *Listeria*, 9.9% *Escherichia/Shigella*, 10.1% *Pseudomonas,* and 6.2% *Enterococcus*.

### 3.2. Fecal Microbiota Diversity in Clinically Healthy Piglets

**Figure 2:**
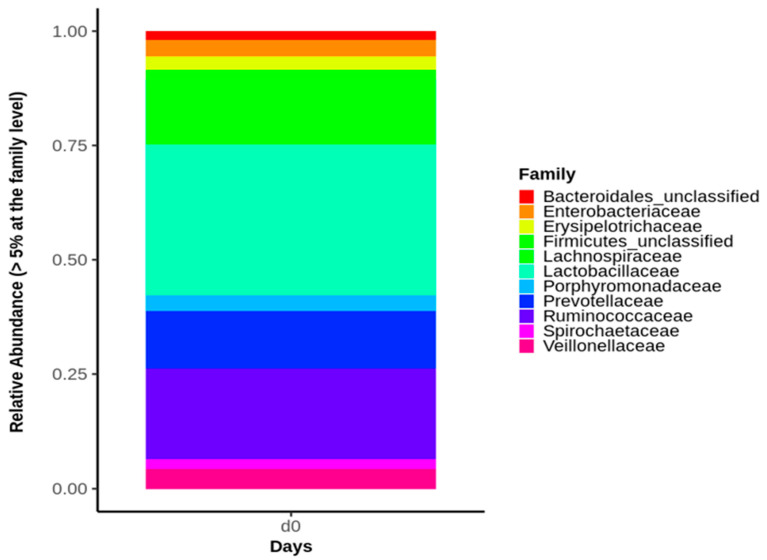
Relative abundance of the major bacterial families identified in the fecal microbiota of the four piglet groups at Day 0 (D0). Only bacterial families representing at least 5% of piglet fecal microbiota are shown.

It is worth noting that prior to the bacterial challenge (D0), none of the pigs in any of the 4 groups showed signs of diarrhea or anorexia or had to be removed from the experiment. At D0, no significant differences were found in any of the alpha diversity (Table 1) or the beta diversity indices between the four piglet groups. Moreover, at D0, the predominant phyla were Firmicutes (67.3% average value), Bacteroidetes (20% average value), and Proteobacteria (6.3% average value) within the four animal groups (Figure S1). Accordingly, the *Lactobacillaceae* family (19.6% average value) dominated the fecal microbiota composition at D0, followed by *Lachnospiraceae* (15.4% average value), *Ruminococcaceae* (15.3% average value), and *Prevotellaceae* (9.2% average value), without significant difference in the four animal groups (Figure 2). The main bacterial genus in the fecal samples of the four groups were represented by *Lactobacillus* (19.7% average value), *Ruminococcus* (8.8% average value), and *Prevotella* (5.40% average value). 

### 3.3. Impact of ETEC: F4 Oral Challenge on Piglet Fecal Microbiota Diversity and Composition

Alpha diversity indices (Observed, the Shannon and the InvSimpson indices) were compared between groups A (challenged untreated) and D (unchallenged untreated) at all time points. In fact, significant differences for all alpha diversity measures (*p* < 0.05) were observed between these two groups (A and D) at D4, D7, and D36 of the experiment (Table 1). 

**Figure 3:**
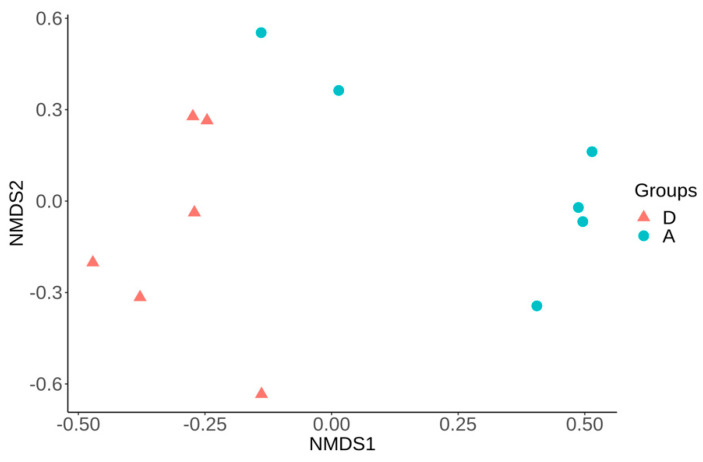
Non-metric multidimensional scaling (NMDS) using Bray–Curtis distance matrix showing significant difference (*p* < 0.05, R^2^ = 0.25) in the fecal microbiota structure between groups A and D at Day 4 (same results were observed at Day 7 and Day 36 (Figure S2)). **A**—challenged untreated group. **D**—unchallenged untreated group.

The analysis of differences in bacterial community per sample compared with all other samples (beta diversity) was based on both Bray–Curtis and Jaccard distance matrices. These analyses showed a significant difference (*p* < 0.05) in the structure and membership community of piglet fecal microbiota at D4, D7, and D36 between group A and D (Figure 3 and Figure S2 (I–II)). Interestingly, at D36, a decrease in *Prevotella* (5% relative abundance) and *Streptococcus* (6% relative abundance) was observed in group A compared to group D. 

**Figure 4:**
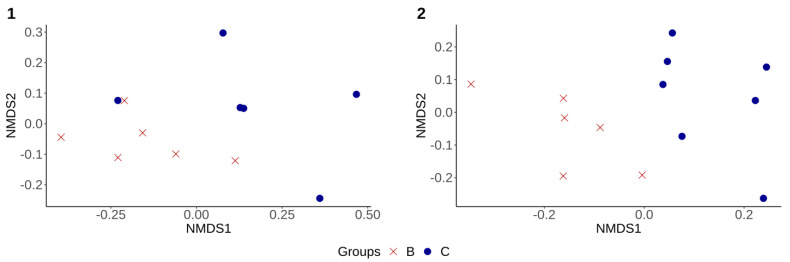
Non-metric multidimensional scaling (NMDS), using Bray–Curtis distance matrix. (**1**) Showed significant difference (*p* < 0.05, R^2^ = 0.13) in the fecal microbiota structure between groups B and C at Day 7. (**2**) Showed significant difference (*p* < 0.05, R^2^ = 0.16) in the fecal microbiota structure between groups B and C at Day 36. **B**—challenged treated group. **C**—unchallenged treated group.

Moreover, alpha and beta diversity analyses were also performed to compare the fecal microbiota diversity in treated groups (B and C) to assess any difference between animals that were treated by CS, whether challenged or not. No significant differences in alpha diversity measures were observed (*p* > 0.05) between these two groups throughout the experiment (Table 1). However, the fecal microbiota structure (beta diversity) of group B was significantly different from that of group C at D7 and D36, respectively (Figure 4 (1–2)). 

### 3.4. Impact of Colistin Sulfate Oral Treatment on Piglet Fecal Microbiota Composition in Healthy or Challenged Animals

Alpha diversity measures were first compared between groups C and D to evaluate the effect of colistin sulfate (CS) on the fecal microbiota of unchallenged weaned pigs. No significant differences were observed in any of the alpha diversity indices between these groups at D2, D4, D7, and D36 (Table 1). Moreover, no significant difference in the structure and membership community of the fecal microbiota (beta diversity) was observed between these groups (C and D) at the same fecal sampling times.

**Figure 5:**
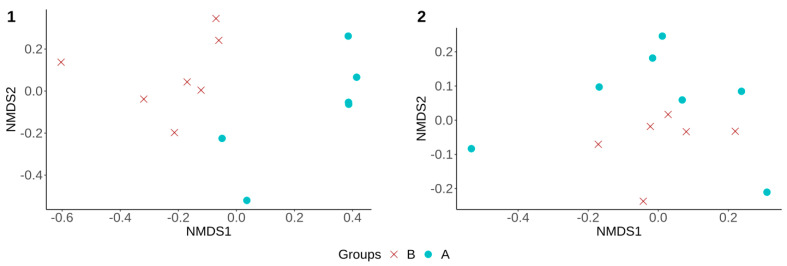
Non-metric multidimensional scaling (NMDS) using Bray–Curtis distance matrix. (**1**) Showed a significant difference (*p* < 0.05, R^2^ = 0.21) in the community structure (beta diversity) between group A and group B at Day 4 (same results were also observed at Day 7). (**2**) Showed no significant difference (*p* > 0.05, R^2^ = 0.1) in the community structure (beta diversity) between the same groups (A and B) at Day 36. **A**—challenged untreated group. **B**—challenged treated group.

Alpha diversity measures were then compared between groups A and B to evaluate the effect of CS on the fecal microbiota of challenged piglets. Shannon and inverse Simpson index values were higher in group A compared to group B, but only at D4 (*p* < 0.05) (Table 1). A significant difference in community structure (beta diversity) between group A and group B was observed at both D4 and D7 of the experiment (*p* < 0.05) (Figure 5-1), with no significant difference at D36 (Figure 5-2). 

**Figure 6:**
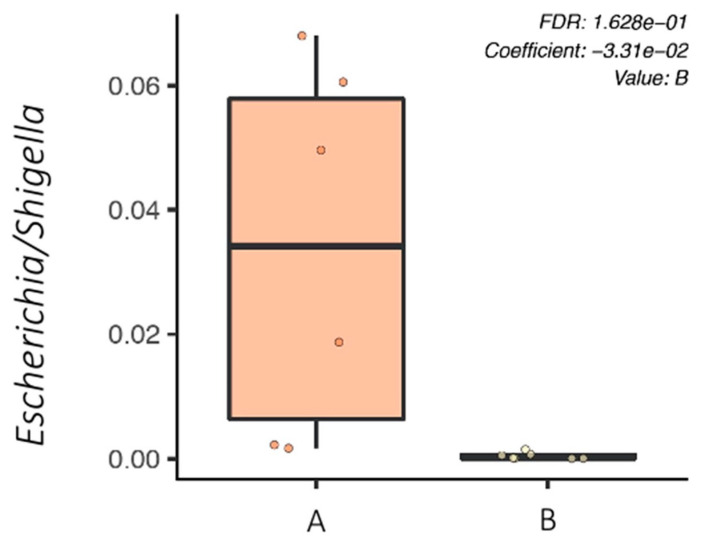
Boxplot showing the relative abundance of the bacterial genus between groups A and B at Day 4 using MaAslin2. At Day 4, the *Escherichia/Shigella* bacterial genus was significantly higher in group A compared to group B. **A**—challenged untreated group. **B**—challenged treated group.

In addition, relative bacterial abundances at the genus level in piglet fecal microbiota were compared between the challenged groups (A and B) using MaAsLin2, which showed that the *Escherichia/Shigella* bacterial genus was significantly higher in group A compared to group B at only D4 of the experiment (Figure 6). Indeed, the relative abundance of the *Escherichia/Shigella* genus was one hundred times smaller in the fecal microbiota of group B compared to that of group A at D4. 

### 3.5. Identification of Specific Microbial Taxa Associated with Oral Challenge or Antimicrobial Treatment 

**Figure 7:**
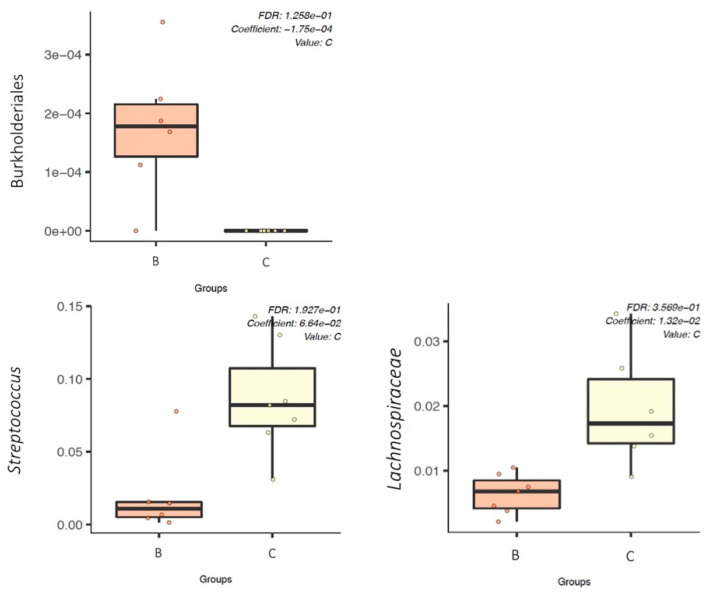
Boxplot showing the relative abundance (using MaAslin2) of the fecal microbiota biomarkers (order of *Burkholderiales*, genus of *Streptococcus*, and family of *Lachnospiraceae*) between groups B and C at Day 36. **B**—challenged treated group. **C**—unchallenged treated group.

A multivariate association with linear models (MaAslin2) was performed in order to determine which microbial taxa were associated with ETEC: F4 challenge or CS treatment in the weaned piglets. Two by 2 comparison between groups (C-D) (unchallenged animals) and (A-B) (challenged animals), respectively, did not reveal any biomarkers associated with CS oral treatment for unchallenged or challenged piglets at D7 and D36. However, Burkholderiales order, including the *Oxalobacteraceae* family, was found to be significantly (*q*-value < 0.05) associated with the ETEC: F4 challenge (group B) compared to unchallenged pigs (group C) at D7 and D36 (Figure 7).

The fecal microbiota in unchallenged treated pigs (group C) was significantly associated with *Streptococcus* genus compared to challenged treated pigs (group B) at D36 (Figure 7). The *Lachnospiraceae* family was also found to be positively associated with unchallenged treated pigs (group C) at both D7 and D36 (Figure 7) compared to group B. In addition, comparison between groups A (challenged untreated) and D (unchallenged untreated) showed that Burkholderiales order was associated with group A, while *Streptococcus* genus and *Lachnospiraceae* family were associated with group D at D36 (Figure S3).

## 4. Discussion

Several studies have focused on the characterization of the gut microbiota in pigs, particularly during the post weaning period, which is associated with considerable economic losses related to post-weaning diarrhea (PWD) episodes. However, studies examining the change in gut microbiota of piglets with clinical ETEC related PWD combined to colistin sulfate (CS) treatment are absent in the scientific literature. The goal of the present study was to bring new insights into the impact of ETEC: F4 challenge and oral CS treatment on the fecal microbiota diversity of both clinically healthy and challenged piglets in a PWD model. We were particularly interested in evaluating how piglet fecal microbiota is impacted by the current practice in the field of using antimicrobials—CS in this case—for metaphylaxis purposes (treating all animals that belong to the same pen/farm whether they are presenting clinical symptoms or are clinically healthy) [36]. It should be stressed here that rectal swab samples used in the present study were collected during experiments performed in previous work [17,30]. In the current study, before the oral challenge, the bacterial communities observed in the fecal microbiota of clinically healthy weaned piglets belonged predominantly to the Firmicutes and Bacteroidetes phylum. Similar to our findings, several previous studies reported that Firmicutes and Bacteroidetes were the two dominant phyla in the fecal microbiota of piglets during the post weaning period [37,38,39,40,41], representing more than 90% of the fecal bacterial community at this stage of a piglet’s life [13]. Additionally, in the present study, the fecal microbiota of clinically healthy piglets, before the ETEC: F4 oral challenge, had high abundance of *Lactobacillaceae*, *Lachnospiraceae*, *Ruminococcaceae*, and *Prevotellaceae*; these results also corroborate previous studies [42,43]. Indeed, these bacterial families, proposed by many authors as a biomarker to predict health status of piglets, are able to metabolize a wide range of complex oligosaccharides and polysaccharides that are involved in preparing a piglet’s intestines and its microbiota to cope with a weaning diet rich in complex carbohydrates compared to the suckling diet [7,15,42].

To the best of our knowledge, our study was the first to demonstrate that clinical PWD caused by an ETEC: F4 challenge that occurs early in the life of a piglet (beginning of the post-weaning period), had a long-term impact on piglet fecal microbiota. Indeed, in the present study, following the ETEC: F4 oral challenge, significant differences in alpha and beta diversity were observed in the fecal microbiota of group A (challenged untreated) compared to group D (unchallenged untreated) at earlier time points (D4 and D7) as well as at D36 post challenge. Other experimental studies have reported that a ETEC subclinical infection did not change the fecal microbiota composition of the challenged weaned piglets [44,45]. This difference could be explained by the fact that in our study most piglets in group A (challenged untreated) developed a profuse diarrhea with altered general condition as well as weight loss as is often observed on farms [17], while in the two other studies, these symptoms were very moderate when present. In addition, different 16S rRNA gene regions were used to study the fecal microbiota, which could be another possible cause for these divergent results [13]. Interestingly, in the current study at D36, there was a significant decrease of *Prevotella* (Bacteroidetes phylum) and *Streptococcus* (Firmicutes phylum) populations in group A compared to group D. It has been reported that both *Prevotella* and *Streptococcus* presence in piglet gut microbiota had a strong correlation with body weight [46], and this finding could be related to the impaired growth observed in group A compared to group D, as reported in our previous study (trial 2) [17]. In fact, other authors have reported that *Prevotella* was positively correlated with luminal secretory IgA concentrations [47]. *Prevotella* is known for its crucial role in metabolizing plant cell wall dietary fiber and thus producing significant amounts of short chain fatty acids (SCFAs) that will subsequently be used by the host as a substrate for metabolic energy production [48]. It is noteworthy that the energy contribution of SCFAs to the basal metabolic rate of pigs is thought to be 30–76% [49]. Although PWD clinical signs had disappeared in group A (challenged untreated) one week after the ETEC: F4 oral challenge, in addition to the fecal shedding of the challenge strain, as previously reported [17,30], the composition of the fecal microbiota remained significantly different in group A (challenge untreated) compared to that of group D (unchallenged untreated) until the end of the experiment (5 weeks later). Moreover, ETEC: F4 oral challenge had a significant effect on beta-diversity indices, whether piglets were treated or not with CS. These findings stress again that ETEC: F4 challenge caused a persistent effect on the composition and diversity of piglet microbiota under the conditions of our study. Knowing that ETEC: F4 colonize the posterior jejunum and ileum segments of piglets, further investigations targeting the bacterial diversity in these segments of piglet intestine are warranted. Such information would refine our knowledge on the effect of this pathogen on the specific microbial communities along the intestinal tract of pigs. The sex of the piglets was not identified in the present study; however, some publications have reported a potential link between the sex of the host and the intensity of changes in the composition and diversity of pig gut microbiota following feed supplementations [50,51]. Therefore, it would be relevant for future studies to investigate any potential links between male or female pigs and the changes that occur in the gut microbiota composition and diversity of these animals, particularly in response to ETEC: F4 challenge.

In the current study, following the CS oral treatment, no significant differences in alpha or beta diversity measures were observed in group C (unchallenged treated) compared to group D (unchallenged untreated), indicating that CS, a narrow spectrum antibiotic, did not significantly alter the fecal microbiota of the treated healthy animals. Our results corroborate those of Fleury et al., who reported that oral CS treatment in healthy piglets, whether given underdosed (50,000 IU/kg BW per day) by oral gavage for 5 days or overdosed (3600 IU/g of feed) for 14 days, was not associated with a significant perturbation in the fecal microbiota of these animals, given the narrow spectrum of CS [29]. However, Li et al., recently reported that the human intestinal bacterial community was significantly disturbed by the combinatorial colistin and amoxicillin (a broad-spectrum beta-lactamase antimicrobial) exposure, illustrating that this combinatorial antibiotic exposure, which may have an impact on several different intestinal bacterial families, could lead to long-term effects on the bacterial diversity of a simulated human intestinal microbiota as well as an increase in the relative abundance of opportunistic pathogens within this microbiota [52]. Likewise, it was reported that the oral administration of broad-spectrum antimicrobials (e.g., amoxicillin, chlortetracycline) in clinically healthy pigs was associated with a significant alteration in the composition of the fecal microbiota of the treated animals [24,53].

In the current study, CS oral treatment was associated with a significant decrease in *Escherichia/Shigella* abundance in group B (challenged treated) compared to group A (challenged untreated) at only D4, indicating that colistin exerts a transient and targeted effect on the fecal microbiota of sick animals during treatment. These results concur with the bacteriological culture results of both fecal ETEC: F4 and total *E. coli* population reported in our previous study (trial 2) [17] and with the findings of Fleury et al., who showed that CS oral treatment in healthy pigs was associated with a significant decrease of the fecal *E. coli* populations during the treatment period, after which these populations rose significantly [29]. These findings are consistent with the fact that CS, a narrow spectrum antibiotic, is effective against Gram-negative bacteria (GNB) and ineffective against Gram positive bacteria, anaerobic bacteria, and mycoplasmas [54]. It has to be noted that in commercial pig herds, CS is mainly administered through water as a therapeutic or metaphylactic treatment for the control of intestinal infections caused by GNB, including PWD [26].

In the present study, the early changes observed in the fecal microbiota due to the ETEC: F4 oral challenge was followed by a long-lasting shift in piglet microbiota compared to the transient effect caused by oral CS treatment, suggesting that the gut microbiota of weaned piglets seem to be resilient to such a perturbation by a narrow-spectrum antibiotic treatment in the conditions of our study. Nevertheless, the identification of the groups of bacteria affected by disruption of the fecal microbiota homeostasis following an ETEC: F4 challenge could serve as a crucial step toward developing feed strategies in order to increase resistance in piglets regarding this pathogen as well as to reduce the use of antimicrobials for the control of PWD in pig production. Consequently, further studies with different approaches, such as metatranscriptomics and metabolomics, are necessary to characterise the role of some gut microbiota subgroups (e.g., *Prevotella* and *Streptococcus*) in piglet health and disease. 

In the current study, the *Burkholderiales* order was associated with sick pigs. Indeed, very few studies have reported the identification of *Burkholderiales* order in pig gut microbiota (mostly in the ileal and colonic microbiota) [45,55], and the role of this bacterial order in diseases as well as in pig zootechnical performance has not been well characterized. However, in a mouse model of anorexia, a positive correlation between the abundance of *Burkholderiales* in mice gut microbiota and low body weight was recently established [56]. This could be why significantly lower weight gain was observed in the challenged piglets compared to the unchallenged piglets in trial 2 of our previous study [17]. In the current study, the *Lachnospiraceae* family was associated with healthy pigs, and this finding concurs with the study of Dou et al., who reported that diarrheic piglets, in natural PWD, had lower abundance of *Lachnospiraceae* in their fecal microbiota compared to healthy piglets [42]. It is noteworthy that given the scarcity of information related to the role of the *Burkholderiales* order and the *Lachnospiraceae* family in the occurrence of PWD, it is crucial to carry out future studies under farm conditions, using a higher number of piglets to further characterise the role of these gut microbiota subgroups in this disease.

Finally, in our study, the fecal microbiota in unchallenged pigs (groups C and D) was significantly associated with *Streptococcus* (a member of the *Lactobacillales* order) at D36. As explained above, the presence of *Streptococcus* strongly correlates with piglet body weight, and it seems that this bacterial genus is associated with healthy microbiota in piglets [46]. These results may serve as an important starting point for future studies into the role of *Streptococcus* (e.g., *Streptococcus faecium*) as a probiotic to improve both the resistance of piglets to bacterial digestive infections and the growth performance of weaned piglets, as it has been shown to be the case for calves [57]. Indeed, developing alternative strategies to antimicrobials is a global concern for the pig industry, particularly in the current context of antimicrobial use reduction in food-producing animals and the growing consumer demand for antibiotic-free breeding. 

## 5. Conclusions

In the present study, the 16S rRNA gene amplicon analysis showed that the community structure of the fecal microbiota of piglets in clinical PWD was strongly influenced by an ETEC: F4 challenge throughout the experiment, whereas the effect of the administration of the narrow spectrum antibiotic CS was limited to the treatment period only. Our results also suggest that there are gut microbiota subgroups of bacterial genus (e.g., *Prevotella* and *Streptococcus*) that are associated with clinically healthy piglets. However, the role of these bacterial genus should be confirmed in future studies using a higher number of animals, preferably under farm conditions. 

## Figures and Tables

**Table 1:** Fecal microbiota alpha diversity indices of the four piglet groups across the five sampling time points.

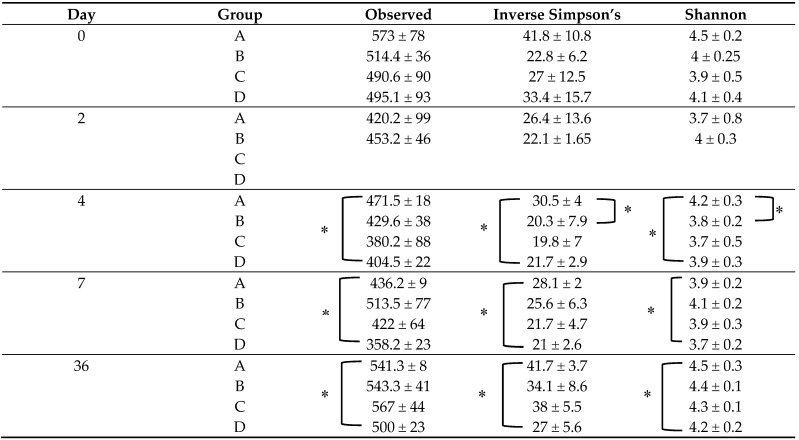

**A**—Challenged Untreated group. **B**—Challenged Treated group. **C**—Unchallenged Treated group. **D**—Unchallenged Untreated group. Rectal swab samples from groups C and D (unchallenged piglets) were not collected at Day 2. * Indicates significant differences (*p* < 0.05).

## Data Availability

The raw sequencing data were deposited in the NCBI Sequence Read Archive under accession number PRJNA724700 (https://www.ncbi.nlm.nih.gov/sra, accessed on 27 April 2021).

## Supplementary Materials

The following are available online at https://www.mdpi.com/article/10.3390/microorganisms9071459/s1. Figure S1: The predominant phyla within the fecal microbiota of the four piglet groups at Day 0. Figure S2: Non-metric multidimensional scaling (NMDS), using Bray–Curtis matrix distance. I- Showed a significant difference (*p* < 0.05, R^2^ = 0.12) in the fecal microbiota structure between groups A and D at Day 7. II- Showed a significant difference (*p* < 0.05, R^2^ = 0.16) in the fecal microbiota structure between groups A and D at Day 36. **A**—challenged untreated group. **D**—unchallenged untreated group. Figure S3: Boxplot showing the relative abundance (using MaAslin2) of the fecal microbiota biomarkers (order of *Burkholderiales*, family of *Lachnospiraceae* and genus of *Streptococcus*) between groups A and D at Day 36. **A**—challenged untreated group. **D**—unchallenged untreated group. Table S1: Composition and relative abundance of the microbial community of the three negative controls (sterile water).
