# Supplementary material for: Evolution of Pig Fecal Microbiota Composition and Diversity in Response to Enterotoxigenic Escherichia coli Infection and Colistin Treatment in Weaned Piglets
Source: Microorganisms. 2021 Jul 7;9(7):1459. doi: 10.3390/microorganisms9071459 (PMC8306681; doi:10.3390/microorganisms9071459)
Supplement: Supplementary file 1 [file microorganisms-09-01459-s001.zip › microorganisms-1231707-supplementary.pdf]

*Supplementary information to the article:*

**Evolution of pig fecal microbiota composition and diversity in response to enterotoxigenic *Escherichia coli* infection and colistin treatment in weaned piglets**

Mohamed Rhouma[1,2*], Charlotte Braley[1,2], William Thériault[1,2], Alexandre Thibodeau[1,2], Sylvain Quessy [1,2], Philippe Fravalo[1,2,3]

[1] Department of Pathology and Microbiology, Faculty of Veterinary Medicine, Université de Montréal, Saint-Hyacinthe, QC, J2S 2M2, Canada; braley.braley@umontreal.ca (B.C.); william.p.theriault@umontreal.ca (W.T.) ; alexandre.thibodeau@umontreal.ca (A.T.) ; sylvain.quessy@umontreal.ca (S.Q.) ; philippe.fravalo@lecnam.net (P.F.)

[2] Groupe de Recherche et d'Enseignement en Salubrité Alimentaire (GRESA), Faculty of Veterinary Medicine, Université de Montréal, Saint-Hyacinthe, QC, J2S 2M2, Canada.

[3] Conservatoire national des arts et métiers (CNAM), 292 rue Saint-Martin, Paris, France.

* Corresponding author: mohamed.rhouma@umontreal.ca. +1 (450) 773 8521 Ext: 52416

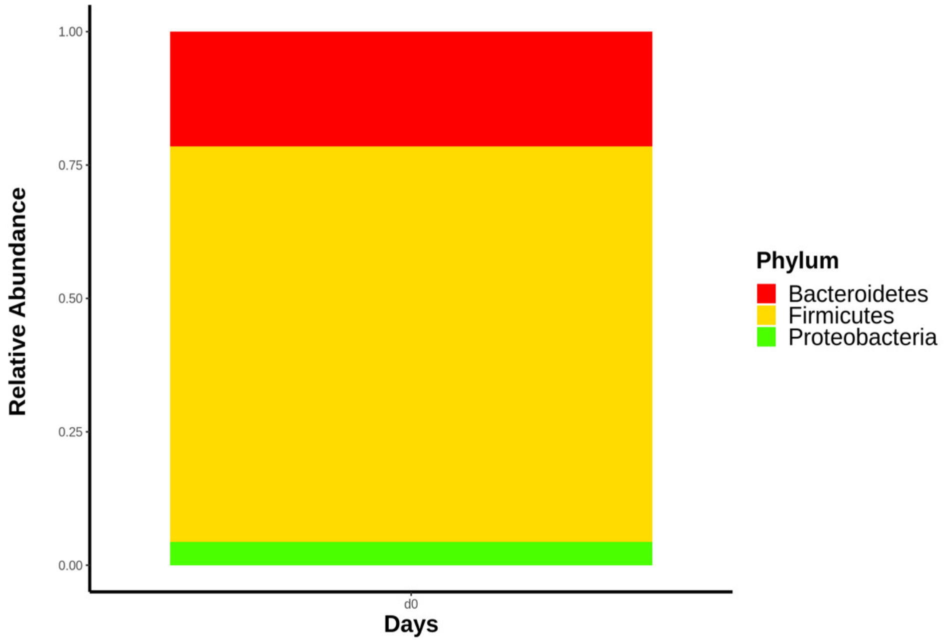


**Figure S1.** The predominant phyla within the fecal microbiota of the four piglet groups at Day 0.

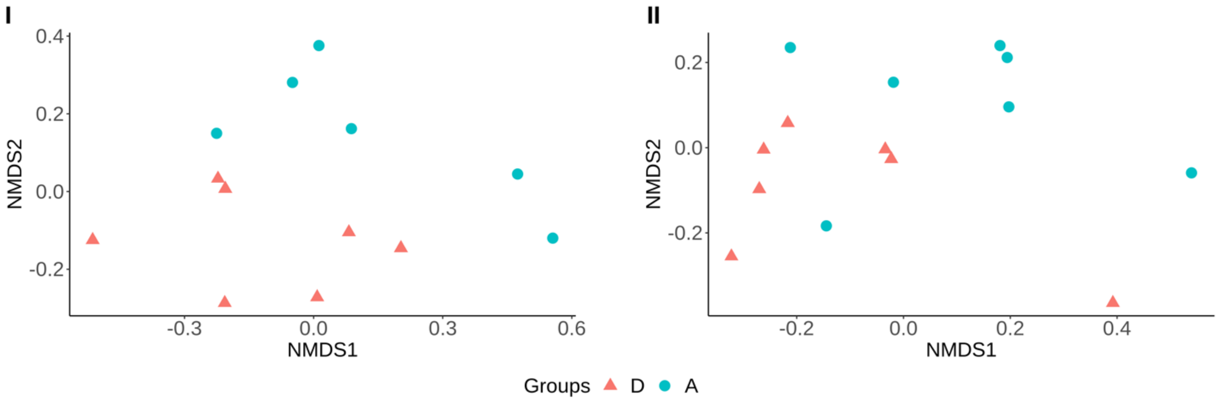


**Figure S2.** Non-metric multidimensional scaling (NMDS), using Bray-Curtis matrice distance. **I-** Showed a significant difference ($p < 0.05$, $R^2 = 0.12$) in the fecal microbiota structure between groups A and D at Day 7. **II-** Showed a significant difference ($p < 0.05$, $R^2 = 0.16$) in the fecal microbiota structure between groups A and D at Day 36. **A** — challenged untreated group. **D** — unchallenged untreated group.

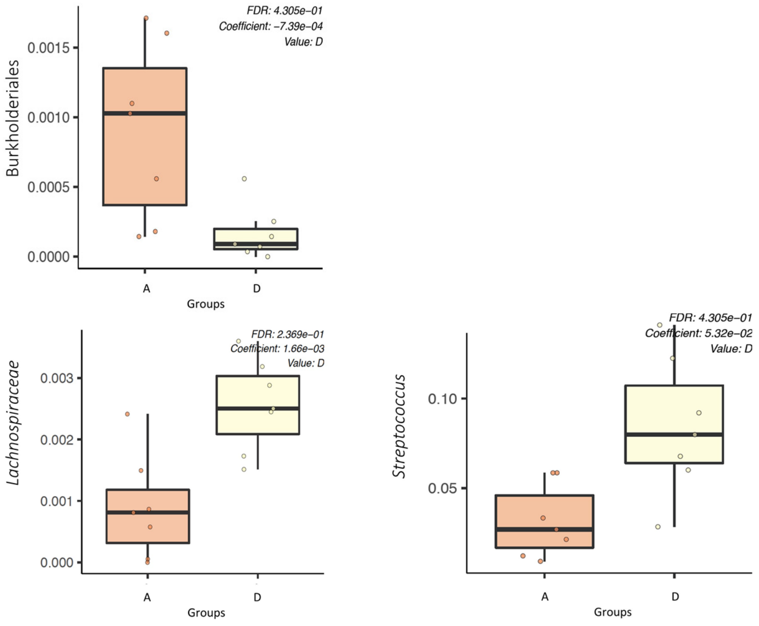


**Figure S3.** Boxplot showing the relative abundance (using MaAslin2) of the fecal microbiota biomarkers (order of *Burkholderiales*, family of *Lachnospiraceae* and genus of *Streptococcus*) between groups A and D at Day 36. **A—** challenged untreated group. **D—** unchallenged untreated group.

**Table S1**. Composition and relative abundance of the microbial community of the three negative controls (sterile water)

| Genus | Abundance relative (%) | Samples |
|---|---|---|
| *Halomonas* | 42.0 | Control 1 |
| *Shewanella* | 32.2 | Control 1 |
| *Prevotella* | 18.7 | Control 2 |
| *Lactobacillus* | 17.1 | Control 3 |
| *Lactobacillus* | 12.9 | Control 1 |
| *Ruminococcaceae_unclassified* | 13,00 | Control 2 |
| *Lachnospiraceae_unclassified* | 10.6 | Control 3 |
| *Pseudomonas* | 9.6 | Control 1 |
| *Streptococcus* | 7.4 | Control 2 |
| *Ruminococcaceae_unclassified* | 5.9 | Control 3 |
| *Roseburia* | 5.8 | Control 2 |
| *Butyricicoccus* | 5.8 | Control 2 |
